# Apoceruloplasmin: Abundance, Detection, Formation, and Metabolism

**DOI:** 10.3390/biomedicines9030233

**Published:** 2021-02-25

**Authors:** Maria C. Linder

**Affiliations:** Department of Chemistry and Biochemistry, California State University, Fullerton, CA 91831, USA; mlinder@fullerton.edu

**Keywords:** ceruloplasmin, apoceruloplasmin, non-ceruloplasmin copper, ceruloplasmin secretion and turnover

## Abstract

Ceruloplasmin, the main copper-binding protein in blood and some other fluids, is well known for its copper-dependent enzymatic functions and as a source of copper for cells. What is generally unknown or ignored is that, at least in the case of blood plasma and serum, about half of ceruloplasmin is in the apo (copper-free) form. This has led to some misconceptions about the amounts and variations of other copper-binding proteins and so-called “free copper” in the blood that might be indicators of disease states. What is known about the levels, sources, and metabolism of apo versus holo ceruloplasmin and the problems associated with measurements of the two forms is reviewed here.

## 1. Introduction

**Figure 1:**
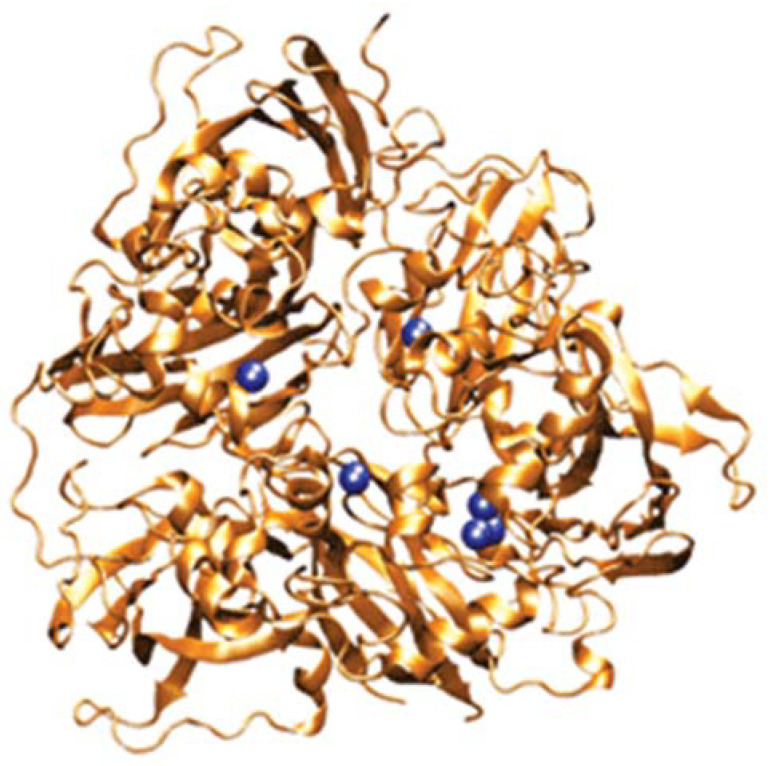
Crystallographic three-lobed structure of human holoceruloplasmin, showing the positions of the 6 internal (integral) copper atoms (dark blue), and based on the studies of Zaitseva et al. [16]. Reprinted with permission from ACS C & E News (8533scicon_cp). Two additional “labile” copper binding sites on the surface of the molecule have also been identified in vitro [17,18,19], although it is unclear that these are normally occupied in vivo.

Ceruloplasmin is a major blood plasma protein, also found in some other mammalian body fluids, including cerebrospinal and amniotic fluids, and milk [1,2]. Blood ceruloplasmin is produced primarily by liver hepatocytes. It has a multiplicity of functions, most of which depend upon that fact that it has six copper atoms buried in its normal, compactly folded state (Figure 1). These copper atoms are the basis of its catalytic activities, which range from oxidizing Fe(II), certain amines, and nitrogen oxide, to neutralizing extra electrons from reactive oxygen species (antioxidant activity) [1,3,4]. It is also a major source of copper ions for uptake by cells throughout the mammalian organism [5,6]. During delivery of ceruloplasmin-copper, apoceruloplasmin (containing no copper) is formed [7]. Ceruloplasmin levels in the blood are dependent upon nutritional copper adequacy. Although excess copper intake fails to increase circulating levels of the protein [1,8], reduced levels of the enzymatically active protein (dependent on copper) are a good indicator of copper deficiency [1,9]. With increasing copper deficiency, there is not only less total ceruloplasmin protein in the circulation, but there are increasingly smaller amounts of the active holo form [9]. Copper is incorporated into ceruloplasmin by the copper “pump”, ATP7B, located in the transGolgi network [4,10,11], after synthesis of the apoprotein and co-translational initiation of its glycosylation in the rough endoplasmic reticulum, followed by modifications/maturation of the glycosyl units in the Golgi apparatus [12,13]. In the absence of functional ATP7B, there still is secretion of ceruloplasmin into the blood plasma, but it is largely or completely in the copper-less apo form [14]. Thus, apoceruloplasmin in the blood can come from at least two sources: (i) the conversion of holo to apo ceruloplasmin during the transfer of copper to cells; (ii) its secretion without copper by exocytosis. Whether apoceruloplasmin per se has a specific function is unknown and has not really been investigated. Ceruloplasmin is removed from the blood by receptor-mediated endocytosis via the hepatocyte galactose receptor, after desialylation by the hepatic endothelial cells [15]. Although not established, it is likely that apo and holo ceruloplasmin are both removed from the circulation in this way (see later).

**Figure 2:**
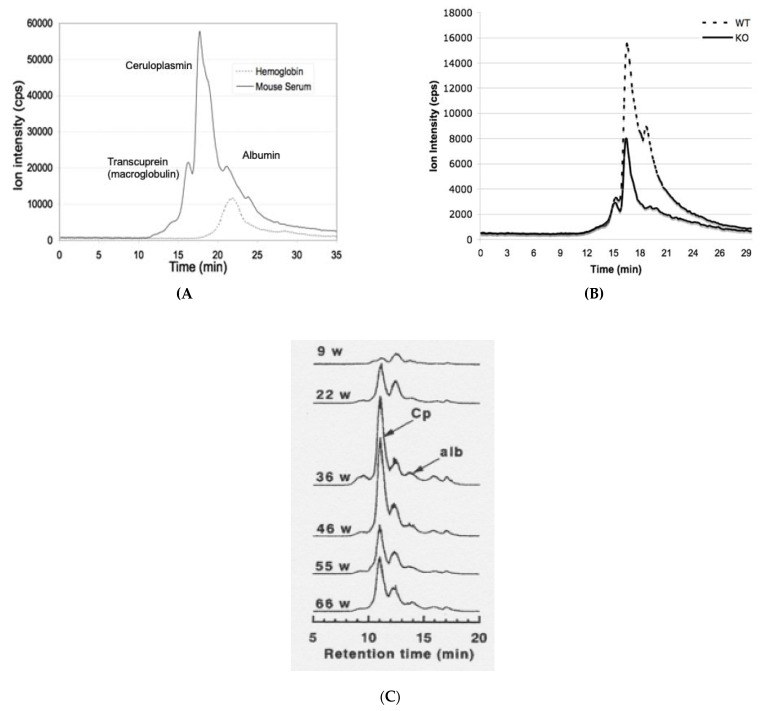
Examples of elution of plasma copper-binding components in large pore size exclusion chromatography detected by tandem ICP-MS, identifying peaks attributable to ceruloplasmin and some other known copper carriers. The x-axis shows elution over time (min); the y-axis is the content of ^63^Cu obtained by mass spectrometry as signal intensity of the ion, counts per second (cps). (**A**,**B**) Biosep 4000 HPLC, also showing mouse serum and the elution of hemoglobin standard, on the left (**A**), and on the right (**B**) mouse serum from normal wildtype mice (WT; dashed line) as well as mice where the ceruloplasmin gene has been knocked out (KO; solid line). The figure in (**A**) modified from one in Cabrera et al. [23]; figure (**B**) is reprinted with permission from Gray et al. [24]. For both, copper profiles were averages for two WT plus or minus three KO mice. Knocking out ceruloplasmin reduced the signal 35%, indicating that ceruloplasmin accounted for 65% of the total serum copper. (**C**) GS-520 HPLC, showing Cu components in serum of Long-Evans Cinnamon (LEC) rats at different ages, in weeks (w), reprinted with permission from Komatsu et al. [25]. This figure was included to further illustrate that there are many copper binding components other than ceruloplasmin in the blood plasma. The article from which it comes investigated how mutations in the copper transporter (ATP7B) of LEC rats and the resultant inflammation (which occurs in mid-life in these rats) affect levels of ceruloplasmin-copper in the blood. The figure shows that proportions of copper attributable to ceruloplasmin are highest during the inflammatory phase.

What has not been adequately appreciated is that most probably in the normal state, apoceruloplasmin may account for about half of the total ceruloplasmin protein in the circulation, as detailed in this review. This has led to some misconceptions about just how much copper in the plasma is due to ceruloplasmin and the meaning of assays for “non-ceruloplasmin” copper (or “free copper”) based on this premise. For non-ceruloplasmin copper assays, the amounts of total ceruloplasmin protein are multiplied by the copper content of holoceruloplasmin. By these calculations, the proportion of plasma copper in ceruloplasmin is greater than 80%, the rest being considered “non-ceruloplasmin copper”. Indeed, the literature (including the clinical literature) is full of statements that ceruloplasmin accounts for 95% of total plasma copper [20,21,22]. Clearly, if a substantial portion of ceruloplasmin in the plasma is in the apo form, the percentage of total copper contributed by ceruloplasmin is much lower. More importantly, it is clear from a substantial body of data that the proportion of total plasma copper attributable to ceruloplasmin is indeed less [3,4], as shown, for example, by elutions of copper components in plasma separated in size exclusion chromatography on large pore gels. Figure 2 shows HPLC chromatograms of Cu peaks eluting from the blood plasma of adult mice (Figure 2A) and rats of different ages (Figure 2B) in terms of weeks, where enzyme activities, immunoassays, and/or apparent molecular weight markers identified where copper-carrying ceruloplasmin, albumin and macroglobulins/transcuprein elute. Clearly, the ceruloplasmin peak predominates but accounts for well under 80–90%.

This review examines the accumulated evidence that apoceruloplasmin is a major portion of total ceruloplasmin in mammalian blood plasma, and discusses problems associated with measurements of the amounts of apo and holo forms of this protein that need to be addressed and might in fact lead to new conceptions about the potential functions and metabolism of the apoprotein in health and disease.

## 2. Evidence for the Presence and Amounts of Apoceruloplasmin in the Blood Plasma

The first evidence that apoceruloplasmin is present in the blood was provided by Holtzman and Gaumnitz in 1970 [9]. They depleted young and growing male rats of ceruloplasmin by placing them on a copper-deficient diet. This resulted in a rapid fall in ceruloplasmin enzyme activity (measured with p-phenylene diamine) to virtually zero over four weeks, compared to rats on normal diets, where activity remained unchanged. Although the plasma of the deficient animals had no oxidase activity, they found that ceruloplasmin was still present in immunoprecipitates and behaved identically in electrophoresis to that of apoceruloplasmin produced by removing copper from the purified protein. The antibody they used was raised in rabbits injected with ceruloplasmin that had been purified to homogeneity. Moreover, their polyclonal antibody bound apo and holo ceruloplasmins equally well. Using quantitative immunoprecipitation, they calculated that the plasma of their copper deficient rats—which had no ceruloplasmin enzyme activity and thus no holoceruloplasmin—contained about one quarter of the total ceruloplasmin protein (holo plus apo) present in their control (normal) rats.

**Figure 3:**
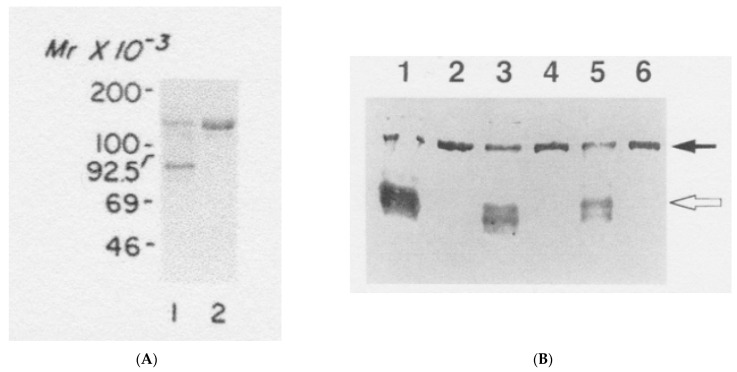
SDS-PAGE of ceruloplasmins without and with heating in SDS-sample buffer prior to electrophoresis, showing separation of two ceruloplasmin bands, when not heated. (**A**) Purified human ceruloplasmin, not heated (lane 1) or heated at 100 °C for 3 min (lane 2), prior to electrophoresis, and stained with Coomassie blue. Migration of molecular weight markers (Mr, in kDa) is also shown on the left. Reprinted with permission from Sato and Gitlin [26]. (**B**) Immunoblot of human ceruloplasmin isolated by immunoaffinity chromatography from secretions of cultured rat hepatic cells, where apo and holo forms were distinguished by not heating the samples prior to SDS-PAGE (lanes 1, 3, 5), and compared with the results from preheating (lanes 2, 4, 6). The sources of the ceruloplasmins were secretions from long-term cultured hepatocytes of Long-Evans Agouti (LEA) and Long-Evans Cinnamon (LEC) rats that had been transfected with human ceruloplasmin cDNA, and ceruloplasmins isolated and immunodetected used monoclonal antibody against human ceruloplasmin that did not react with rat ceruloplasmin. Reprinted with permission from Nakamura et al. [14]. The objective of the Nakamura studies was to determine how a lack of the ATP7B copper pump in LEC hepatocytes—compared to the normal LEA rats—would affect synthesis and the secretion of apo and holo ceruloplasmin produced by the human gene transfected into these cells.

In 1991, Sato and Gitlin reported that after purification from human plasma, ceruloplasmin contained two protein bands in SDS-PAGE gels when not subjecting samples to the usual heat pretreatment (100 °C for 3 min) in reducing the SDS sample buffer (Figure 3A, left) [26]. Heating proteins in SDS with mercaptoethanol (reducing internal disulfide bonds) results in the complete unfolding (denaturation) of proteins into strands coated with SDS and gives them similar charge/mass ratios so they separate only on the basis of size. Thus, their native charge and shape or folding plays no role. Without heating, some proteins will not denature in SDS, and thus maintain their folded state and perhaps also some of their native charge. This appears to be the case for one form of ceruloplasmin, which we know from its X-ray structure is a very compact protein and would thus migrate through gel pores more easily and rapidly than its denatured form, explaining the lower of the two bands observed in these studies (Figure 3A, lane 1). The two Coomassie blue-stained bands separated well in a 7.5% acrylamide resolving gel, and were present in about 30 to 70% proportions, with fewer of the slower migrating species. The faster moving band (undenatured ceruloplasmin) migrated similarly to the upper band when the sample was preheated with the reducing SDS sample buffer, as is normally done for SDS-PAGE, and this single band had the apparent molecular weight (~130 kDa) predicted for ceruloplasmin. The faster migrating band obtained without heating the sample had ceruloplasmin oxidase activity, implying that it contained copper. Although not shown, they stated that both bands reacted with antibody against ceruloplasmin. Importantly, isolating ^67^Cu-ceruloplasmin from rats pretreated with the radioisotope, they found that ^67^Cu was only in the faster migrating band, confirming that this was holoceruloplasmin and that they had found a way of separating the apo and holo forms of the protein. Also important is that they found no copper associated with the apoceruloplasmin band. Together, their findings indicated that normal human plasma contains both apo- and holoceruloplasmin; that the two forms purify together using the procedure established by Putnam’s group [27]; and that levels of the two forms were not that different. The same procedure (SDS-PAGE, without and with heating) was later applied by Nakamura et al. in 1995 to ceruloplasmin secreted by rat hepatocytes, after transfection with human ceruloplasmin cDNA (Figure 3B) [14]. In this case, detection was by immunoblotting, and the holo form clearly predominated.

**Figure 4:**
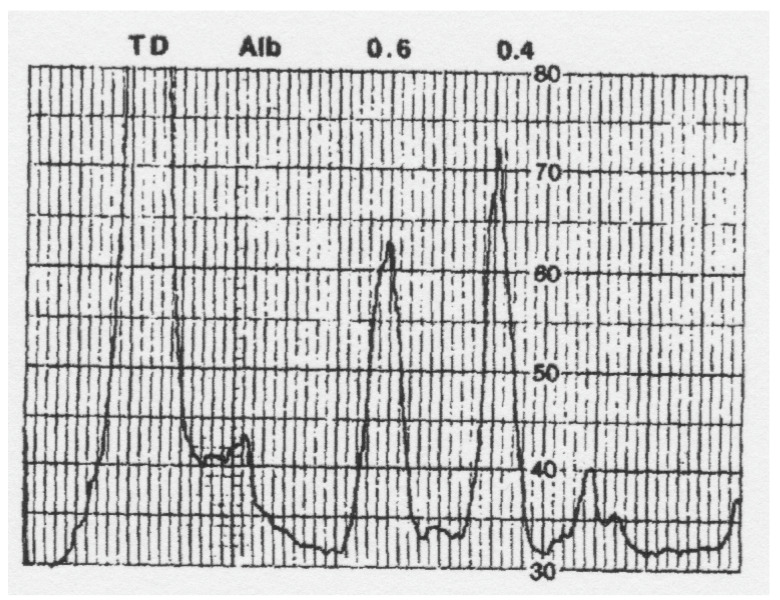
Native (non-denaturing) PAGE, in tube gels, of semi-purified rat ceruloplasmin, as detected by UV absorbance scanning of the gel at 280 nm. The top of the gel is at the right; the bottom, showing the tracking dye (TD), is on the left. Some albumin (Alb) was still present. Holo and apo ceruloplasmins migrated with Rfs of about 0.6 and 0.4, respectively. The resolving gel was 5% polyacrylamide, resolving buffer pH 8.8. Reprinted with permission from Middleton and Linder [28].

Similar results to those of Sato and Gitlin [26] were obtained for rat ceruloplasmin by our laboratory in 1993 [28]. In this case, ceruloplasmin was partially purified by a different procedure (DEAE chromatography in acetate, pH 5.5, plus preparative non-denaturing/native polyacrylamide tube gel electrophoresis). The resulting material separated into two bands with relative migration (Rf) values of 0.4 and 0.6. A UV absorbance (A_280_ scan) of the native PAGE tube gels (Figure 4) shows almost equal amounts of 280 nm absorbance for the two bands, implying that, at least in these rats (which had been treated with estrogen), the concentrations of the apo and holo ceruloplasmin forms were about the same. About half of the ceruloplasmin was in the apo form. As in the case of the Sato and Gitlin results [22], there was a marked difference in migration of the apo and holo ceruloplasmin bands, the holoceruloplasmin migrating significantly more rapidly. This implies that the holoform of the protein is very compact—also indicated by the X-ray structure, while the apo form is much more loosely structured, thus hindering its progress through the gel.

**Figure 5:**
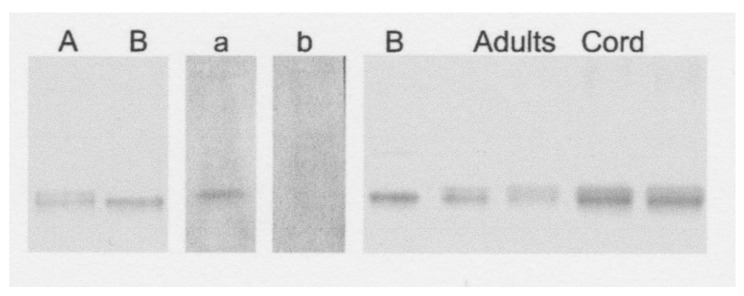
Native PAGE of human serum before and after removal of most of the copper by dialysis against NaCN, to form apoceruloplasmin. Left to right: immunoblot, with antibody against ceruloplasmin, A—before, and B—after Cu removal; in situ staining for ceruloplasmin oxidase activity, a—before, and b—after NaCN dialysis; immunoblot of apoceruloplasmin-rich plasma (B), 2 lanes each of normal adult human plasma and cord blood. Gel was 8% acrylamide, and resolving buffer was pH 7.4. Reprinted with permission from Hirano et al. [29].

Subsequent analyses by Hirano et al. [29] of apo and holo forms of ceruloplasmin from subjects with Wilson disease and normal controls also used native PAGE. In Wilson disease, the transport protein in the transGolgi network that provides copper to ceruloplasmin (ATP7B) is dysfunctional to various degrees. Thus, much less or no copper is available for formation of the holo protein. Hirano et al. used a different form of native PAGE than in the Linder lab studies. The resolving gel was 8 vs. 5% acrylamide, and the pH for resolution was 7.4 vs. 8.8. This led to a much poorer separation (Figure 5). In addition, they detected the ceruloplasmin forms by immunoblotting. Although somewhat difficult to see, the results in Figure 5 show a single distinct protein band for apoceruloplasmin after treating the purified protein to remove copper (lanes B). This contrasts with the lanes for adult human plasma and cord blood, where two almost-overlapping bands are detected, presumably corresponding to the apo and holo forms. Densitometry applied to the immunoblots for nine Wilson disease patients and twelve control sera indicated that holoceruloplasmin was about half of the total (46%) in the controls (range 40–56%), while the percentages for Wilson disease patients varied from about 8% (considered negligeable) to about 40%, in five patients ages 4–12, and three patients, ages 12–18 years of age, respectively.

**Figure 6:**
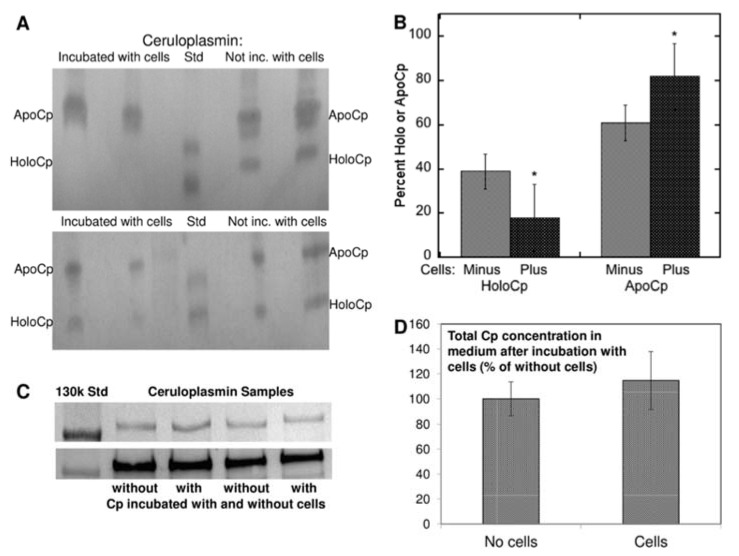
Ratios of apo to holo ceruloplasmin (Cp) in rat plasma, and effects of incubating purified rat ceruloplasmin with cultured cells. (**A**) Two examples of immunoblots of ceruloplasmin incubated without cultured cells (right two lanes), and with cultured cells (left two lanes), showing decreases or elimination of holoceruloplasmin by exposure to cells, and detecting the two forms by immunoblotting after native PAGE in 4.5% gels, with pH 8.8 resolving buffer. (**B**) Summarized densitometry data for multiple experiments of the type shown in (**A**), indicating loss of holo Cp and gain in apoCp during incubation with cells; * *p* < 0.01 for difference. (**C**) Evidence that total Cp levels did not change during incubation with cells. (**D**) Summarized densitometric data for experiments such as those in (**C**). Reprinted with permission from Ramos et al. [7].

Further information on the origins of apoceruloplasmin and its abundance relative to holoceruloplasmin was obtained in our laboratory, in experiments investigating the means by which copper in ceruloplasmin is taken up by cells [7]. For these studies, we isolated ^67^Cu-labeled ceruloplasmin secreted by cultured human HepG2 cells or from the plasma of mice injected with this radioisotope, and followed uptake of the radioactive copper by cultured human and mouse cells, in the absence and presence of various inhibitors. It is well established that copper in holoceruloplasmin is buried within its compact structure (Figure 1) and cannot be dialyzed away or removed by exposure to chelating agents such as EDTA, unless first denatured. However, in these studies, the ^67^Cu in ceruloplasmin was readily taken up by various cell types in tissue culture (Figure 6). During incubation of the cells with the ^67^Cu-labeled ceruloplasmin for 3 h or more, the levels of ceruloplasmin protein in the medium did not change (as determined by immunoblotting; Figure 6C,D). However, the proportion of apoceruloplasmin in the medium markedly increased (Figure 6A,B). In one of nine separate experiments, holoceruloplasmin even completely disappeared (Figure 6A, top), with a concomitant increase in the apo form. Thus, uptake of copper from holoceruloplasmin resulted in its conversion to copper-less apoceruloplasmin. Uptake was not prevented by inhibitors of endocytosis, but was prevented by blocking its reduction from Cu(II) to Cu(I) by an excess of Fe(III), which is reduced by the same cell surface reductases [30,31]. Release of copper from ceruloplasmin did not occur in the absence of cells but only when cells were present. Moreover, because it was not taken up by endocytosis, it must have occurred at the surface of the cell plasma membrane, presumably due to conformational changes in ceruloplasmin induced by interaction with transporters and/or reductases in the cell membrane. These findings imply that the delivery of copper to cells by ceruloplasmin is a major way in which apoceruloplasmin is produced and circulates in blood and interstitial fluid. Indeed, it may be the most important way it is formed, and the main reason for the abundance of apoceruloplasmin in the blood.

**Figure 7:**
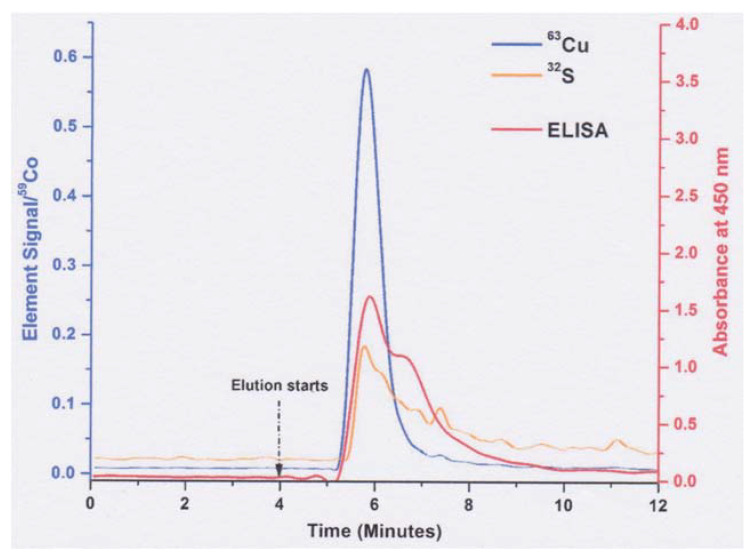
Immunoaffinity chromatography of human ceruloplasmin, coupled with ICP-MS to detect copper (blue line) and ELISA immunoassays to quantify ceruloplasmin protein (red line). Diluted blood serum was applied to the column to bind with the immobilized antibody against human ceruloplasmin, and proteins not binding to the antibody washing through. The graph shows what occurs when elution buffer is applied (glycine buffer, pH 2.2). The signal for ^63^Cu is given relative to the ^59^Co standard (left y-axis); ELISA absorbance values are on the right y-axis. The orange line is ^32^S, which reflects the elution of proteins in general. Reprinted without changes, from Bernevic et al. [32], via the Creative Commons license (http//creativecommons.org/publicdomain/zero/1.0/ (accessed on 30 December 2020)).

Additional suggestive evidence of relative apoceruloplasmin abundance is shown in the report of Bernevic et al. [32], where human serum was applied to immunoaffinity chromatography of human serum coupled to ICP-MS and ELISA assays of ceruloplasmin protein. Serum was applied to a column on which antibody raised against human ceruloplasmin in eggs had been immobilized, which would result in binding of ceruloplasmin to the column, allowing other proteins to wash out. Then, an elution buffer (pH 2.2 glycine) was applied to release the antibody-bound material (ceruloplasmin). Figure 7 shows the elution of copper (blue line) and ceruloplasmin protein (red line). The two main copper and protein peaks coincide well; however, there is a major “shoulder” on the ceruloplasmin protein peak, eluting later, which has little or no copper, and which the authors attribute to “Cp fragments that do not contain an copper”. There is no evidence that significant amounts of ceruloplasmin fragments are normally in the blood plasma, and this peak has ceruloplasmin protein and apparently no copper; therefore, the most reasonable explanation is that it is apoceruloplasmin. If so, examining the data suggests there is perhaps twice as much holo- as apoceruloplasmin. However, because antibodies (in the column as well as in the ELISA test for ceruloplasmin protein) will have variable affinities for the two forms of the protein, one cannot be certain of their actual relative abundance.

In summary, these various studies indicate that there are just two main forms of ceruloplasmin: an apo form, with no copper and no enzyme activity, and a holo form, with copper and enzyme activity. There is little or nothing in between the two bands on the gels—no partially metallated intermediates but “all or none” in terms of copper content. Moreover, the copper in ceruloplasmin not only is the basis for its enzyme activity and metal delivery, but brings portions of the protein together to form the symmetrical compact structure of holoceruloplasmin.

## 3. Problems Associated with the Quantitation of Apo and Holo Ceruloplasmin by Immunoassay

Levels of ceruloplasmin in body fluids are measured primarily by immunoassays, and chiefly nephelometry in the case of clinical analyses. The two forms of the protein are very different in their folding; therefore, one might expect that the antigenic portions would also differ, and the mixture of antibodies raised would include some that are unique to one or the other form. Thus, any given polyclonal antibody mixture would be expected to have a different overall affinity for apo versus holo ceruloplasmin. To further illustrate this point, non-immunoassay-based results described in the previous section led to the conclusion that the proportions of apo and holo ceruloplasmin in normal blood fluid are about the same [26,28], while those based on immunoblotting greatly varied [7,14,29], as, for example, with the mouse ceruloplasmin data (Figure 6), where levels of apoceruloplasmin seemed considerably higher than those for holoceruloplasmin (right side of Figure 6A). Whether that is actually the case, however, is unknown, because as already stated polyclonal antibodies for any protein can vary enormously from one batch to another, and are likely to have variable affinities for the two forms of ceruloplasmin.

**Figure 8:**
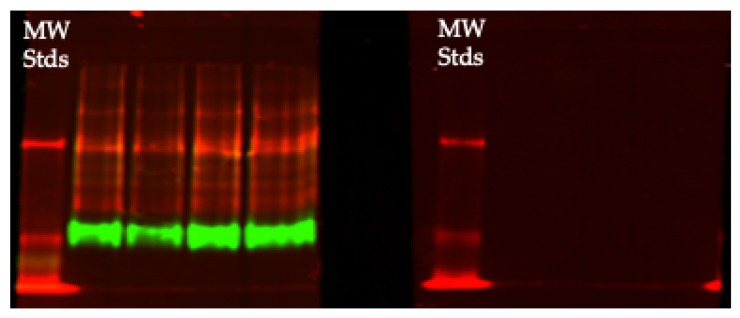
Native PAGE separation of apo (red) and holo (green) ceruloplasmin of rat plasma detected on immunoblots by two separate primary antibodies: against human (green fluorescence; Dako) and rat ceruloplasmins (red fluorescence, in-house) produced in rabbits and goats, respectively, with fluorescent secondary antibodies (donkey anti-rabbit and anti-goat) from Li-COR. Molecular weight standards (MW Stds; upper and lower red bands) were 130 and 100 kDa, but cannot determine molecular weight of the substituents, because this is non-denaturing PAGE where separation depends upon the inherent charge, mass and shape of a protein. Samples are from 4 different rats [33].

In our laboratory, we have tested many commercial polyclonal antibodies raised against human and other forms of ceruloplasmin, for general use in tracking total levels of this protein in denaturing SDS-PAGE, as well as testing for the apo and holo forms, within and across species. Usually, polyclonal antibodies will detect both apo and holo ceruloplasmins by immunoblotting (as, for example, in Figure 6A). However, in some cases they will only detect one of the forms. An example of the latter is shown in Figure 8. Here, we had found one antibody (raised against human ceruloplasmin) that only detected the holo form of rat ceruloplasmin (green fluorescence), and another (raised against rat ceruloplasmin) that only detected the apo rat form (red fluorescence).

To overcome these problems, commercial ceruloplasmin immunoassays are calibrated with known amounts of purified ceruloplasmin protein. However, the purified ceruloplasmin standards most probably contain not only holo but also apoceruloplasmin. Thus, unless the proportion of apo to holo of the samples to be tested is the same as in the standards and the antibodies bind equally well to both forms, how could the actual total content of ceruloplasmin protein be accurately determined?

One way would be if it were first demonstrated that the antibody mixture measured the apo and holo forms equally well. This is what Holtzman and Gaumnitz did when they demonstrated the presence of apoceruloplasmin in the plasma (see earlier) [9]. An obvious alternative might be to produce monoclonal antibodies that either work equally well with both forms, or find separate monoclonals that only bind the apo or holo forms and measure those separately. Indeed, that might lead to some interesting new findings about the metabolism of ceruloplasmin in various normal and abnormal physiological conditions.

## 4. “Non-Ceruloplasmin Copper” Assays

As already indicated in the Introduction, several assays have been developed and applied to blood samples from individuals with various diseases that are based upon the concept of measuring “non-ceruloplasmin copper”. For this, the concentration of ceruloplasmin protein, determined by immunoassay, is multiplied by the percentage of ceruloplasmin mass attributable to copper, and that is subtracted from the total copper in the blood plasma [34,35,36,37,38]. For example, a well-regarded clinical chemistry handbook indicates that the mean concentrations of ceruloplasmin protein in adult human plasma are about 270 mg/L [22]. This value is multiplied by the copper content of holoceruloplasmin (mass/mass), which is about 0.32% (6 × 64 Da for Cu/120,000 ceruloplasmin protein in g/moL). This gives about 860 ug Cu/L. Total plasma copper is about 1000 ug Cu/L, in which case ceruloplasmin Cu would be 86%. (If the two surface labile sites on ceruloplasmin (see Figure 1) are also occupied, this would mean 8 × 64/120,000, or about 0.43%, giving > 1161 ug Cu/L, which is higher than the total in plasma). Indeed, the literature (including the clinical literature) is full of statements that ceruloplasmin accounts for 95% of total plasma copper, and this is particularly attributable to reviews on ceruloplasmin published in the 1990s and later [20,21]. Clearly, if a substantial portion of ceruloplasmin in the plasma is in the apo form, the percentage of total copper contributed by ceruloplasmin is much lower. In fact, other evidence for humans and rodents shows it to be more like 50–70% of the total [3,4,11]. An example is shown by the data in Figure 2A, where one sees a major peak in the center of the elution profile for copper from size exclusion chromatography, with two large “shoulders”, an additional peak towards the farther end, and only a trace in very small components. Similarly, in Figure 2B, the major peak (for ceruloplasmin copper; Cp) is accompanied by many others, particularly eluting later and thus being smaller in size. Therefore, although levels of non-ceruloplasmin copper measured in the way described may have some value, if they show a consistent relationship to a disease or other physiological condition, a variety of copper binding protein and perhaps non-protein components contribute to the pool of “non-ceruloplasmin” copper, and these may independently vary in quantity in different states.

It is worth noting, however, that certain approaches to the quantification of “non-ceruloplasmin copper” currently used are more specific. This is the case for the measurements of what was originally termed “free copper” in blood plasma by Squitti and collaborators [39], and later referred to as “non-ceruloplasmin copper” [40], because “free copper ions” do not occur in living organisms except under highly unusual conditions [41]. The best example is the assay developed by Squitti et al. that has been used to help in the diagnosis of Alzheimer’s disease [40,42]. In this case, the plasma is ultrafiltered through a small pore size exclusion gel, and the Cu(II) in the ultrafiltrate is quantified using a fluorescent probe [40]. Levels of this kind of low molecular weight copper are increased in Alzheimer’s disease subjects. Being of very low molecular weight, what is being measured there may be the small copper carrier that has been detected in the urine [43] or blood plasma [11] of Wilson disease model mice.

## 5. What Determines the Levels of Apoceruloplasmin in Blood Fluid

The levels of anything in the blood are the result of the rate of entry minus the rate of removal. Entry would be the secretion of ceruloplasmin into the blood, mainly from hepatocytes [14,26,44], but possibly also from some other cells in certain conditions, such as in inflammation, where cells derived from monocytes (neutrophils, macrophages, and others) become activated to secrete the protein [20,45,46,47]. The existing data from rats and cultured cells indicate that not just the holo but also the apo form of ceruloplasmin is secreted into the blood by hepatocytes. Working with normal and highly copper deficient rats, the latter of which had almost only apoceruloplasmin in the circulation, Holtzman and Gaumnitz saw no difference in the rate of incorporation of ^3^H-leucine into total plasma ceruloplasmin over 40–120 min, indicating (i) that apoceruloplasmin was secreted, and (ii) that its rate of synthesis was identical to that of holoceruloplasmin—which was a portion of the total ceruloplasmin produced by the normal (copper adequate) rats [9]. Our data on the rates of ^3^H-leucine incorporation into the total holo- and apoceruloplasmin over 3 h, for a given volume of plasma separated by non-denaturing electrophoresis (Figure 4) also showed no difference [28]. In vitro studies with cultured hepatic cells confirmed that not only holo but also apo ceruloplasmin can be secreted by these cells [14,26,44].

The main route of removal of ceruloplasmins from the circulation is most probably also the liver. Multiple studies in the 1970s and 1980s, tracing the paths taken by intravenously infused ceruloplasmins in rats (the protein being radiolabeled), indicated that hepatocytes are the main (or perhaps only) site for removal of this protein from the circulation [48]. Prior removal of sialic acid from the purified infused ceruloplasmin increased the rate of removal [49]. The site of sialic acid removal was pinpointed as occurring in the hepatic endothelial cells that line the liver sinusoids [15]; and hepatocyte uptake was shown to be by the galactose receptor, which is involved in the endocytotic uptake of other plasma glycoproteins [50,51,52]. Indeed, immunoreactive ceruloplasmin and its fragments (including some with copper) have been found in the bile itself, which is made by hepatocytes and is the main route for copper excretion in mammals [11]. Whether apo and holoceruloplasmin are both removed primarily or entirely by the liver has not been specifically examined, but seems highly likely because holoceruloplasmin is transformed into apoceruloplasmin while donating its copper to cells, and so the holoceruloplasmin administered intravenously in these studies would have undergone this transformation.

**Figure 9:**
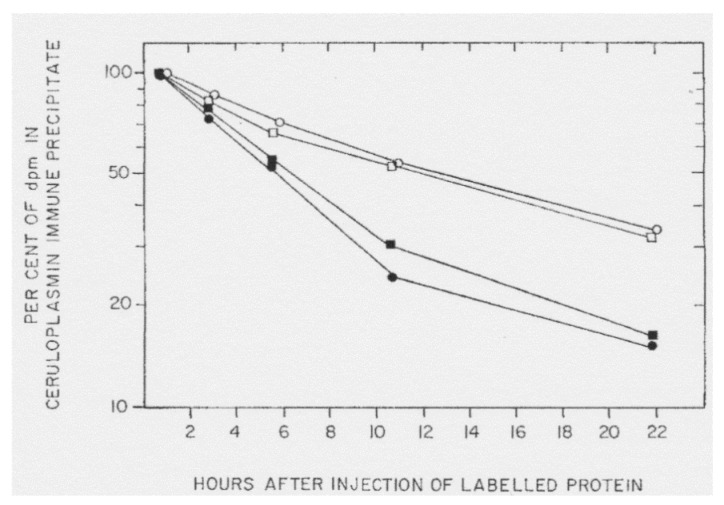
Rates of removal of apo and holo ceruloplasmin from the circulation of copper-deficient rats, after intravenous injection of radiolabeled ceruloplasmins that were relatively rich and relatively poor in the apo form. The ^3^H-ceruloplasmin isolated from a copper-deficient rat (rich in apo ceruloplasmin) was infused intravenously into two copper-deficient rats (dark square and circle), and ^3^H-radioactivity in immunoprecipitates of the plasma samples was measured at different times thereafter. The ^14^C-labeled ceruloplasmin isolated from normal rats was infused into the same kinds of copper-deficient rats, for comparison (clear squares and circles). Reprinted with permission from Holtzman and Gaumnitz [53].

**Figure 10:**
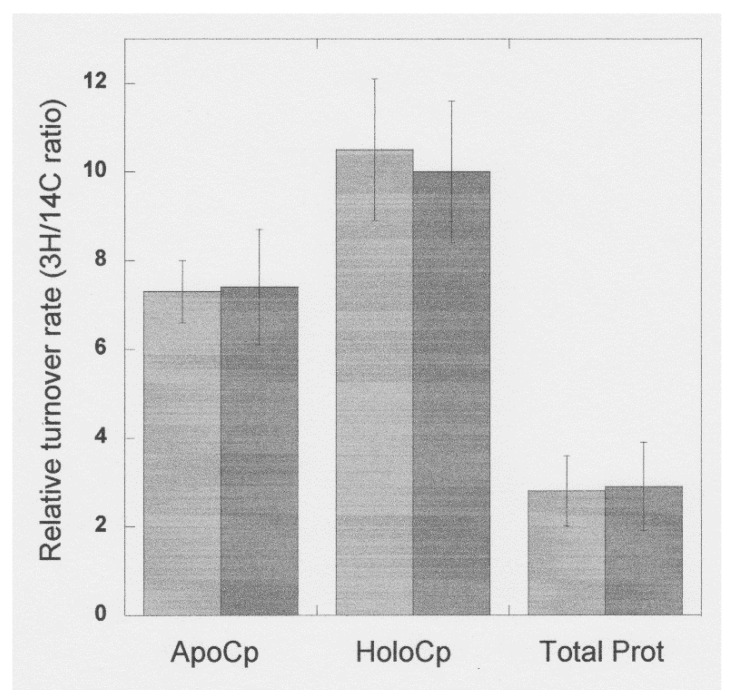
Relative rates of removal of apo and holo ceruloplasmin from the circulation, after synthesis in rats untreated and treated with estrogen, determined by double-labeling. Rats were injected with ^14^C-leucine, followed by ^3^H-leucine 17 h later, and euthanized 3 h later, following which their plasma ceruloplasmin was partially purified, separated in native PAGE tube gels (see Figure 4), and the radioactivity of both isotopes was determined in the apo and holo ceruloplasmin (Cp) gel bands, as well as in the total protein of blood plasma. Dark bars were for rats pretreated with 17β-estradiol for 14 days; light bars control rats only treated with vehicle. Data are means ± SD for groups of 5–10 animals. * *p* > 0.05 for the effect of estradiol. Data are from Middleton and Linder [28].

One conclusion related to the early rat studies of Holtzman and Gaumnitz on apoceruloplasmin (see earlier), was that the turnover of apoceruloplasmin (its removal from the circulation) was more rapid than that of the holo form [53]. In these studies, ceruloplasmin was separately isolated from the plasma of normal and copper-deficient rats (the latter with about 20% of normal ceruloplasmin enzyme activity). These rats had been preinjected with either ^3^H or ^14^C-labeled leucine to radiolabel the proteins. The resulting pure radiolabeled ceruloplasmins were given intravenously to two pairs of copper-deficient rats, and the loss of radioactivity in the immunoprecipitates of their plasma was followed over time (Figure 9). The data (from only four rats) show that the half-life of the infused ceruloplasmins was clearly different, being approximately 2.5-fold more rapid in the case of the ceruloplasmin that had a much lower proportion of the holo form. However, as they freely acknowledge, their data and calculations did not take into account the relative and unknown amounts of holoceruloplasmin in the ceruloplasmins from the normal and copper-deficient rats that had been infused, nor the inherent concentrations of apo and holo in the recipient rats, which are needed to accurately calculate rates of turnover. In addition, turnover was measured in copper-deficient rats, where turnover rates would be expected to differ from those of copper sufficient animals (therefore, these findings are preliminary at best). The half-lives of the infused “more apo” versus “more holo” ceruloplasmins were 6 and 12 h, respectively, and the 12 h value—for the “more holo” form agrees with data from a study by Marceau and Aspin [54], who calculated a half-life of 13 h after infusing normal rats with normal ceruloplasmin (which was probably half in the apo form). Later studies by our laboratory on the effects of estradiol on ceruloplasmin synthesis and turnover [28], indicated that turnover rates of the two forms were virtually identical. Using the double labeling approach of Arias et al. [55], rats were injected with ^14^C-leucine 17 h before the ^3^H-leucine and euthanized 3 h later. This allowed relative measurements of both rates of synthesis and degradation, higher ratios of ^3^H/^14^C reflecting higher rates of turnover, more ^14^C-protein having been degraded/removed during the time interval between the two injections. Radioactivity in apo and holoceruloplasmins was determined after their separation in tube gel electrophoresis (as in Figure 4) and were more or less identical (Figure 10). However, the double-labeling approach does not allow calculation of actual turnover rates, unless exactly the same doses of radioactive amino acid and the efficiencies of their counting are also known—which was not the case. Thus, in normal rats, removal of apo and holo ceruloplasmin from the circulation may be by the same mechanism, involving desialylation and uptake by the galactose receptor, but warrants further investigation.

What we know so far is thus: both the transfer of copper to cells from ceruloplasmin and secretion of the apo protein contribute to the relatively large amounts of apoceruloplasmin found in the blood plasma. Release of apoceruloplasmin into the blood by the secretory pathway even under normal conditions implies either that production of the holo protein is inefficient, and/or that apoceruloplasmin may have some as yet unknown function(s) that remains(s) to be identified.

## 6. Summary and Conclusions

In summary, although much remains to be explored, the best evidence available at this time suggests that levels of apoceruloplasmin in the blood plasma and serum of humans and rodents are close to half of the total ceruloplasmin protein present. The apoceruloplasmin present is the result not only of the release of copper from the holo form during delivery of this trace element to cells, but also from the secretion of apoceruloplasmin by hepatocytes and possibly other cells in some cases. Rates of synthesis and secretion of the holo and apo forms into the blood appear to be similar, and the same may normally be the case for their rates of removal, which occurs at least mainly through endocytosis by hepatocytes via the galactose receptor, after desialylation by hepatic endothelial cells. Apoceruloplasmin concentrations in blood plasma are substantial, and there are many other copper-binding components in the blood beyond ceruloplasmin (which may account for only 50–70% of the total copper); therefore, the development of clinical methods to aid in the diagnosis of physiological and disease changes should not be based on “non-ceruloplasmin copper” and needs to be revisited, taking these matters into account. The fact that not only holo- but apoceruloplasmin is probably normally secreted into the blood suggests that it might have functions independent of those for the holo form, which needs to be further explored.
